# Work-loss years among people diagnosed with diabetes: a reappraisal from a life course perspective

**DOI:** 10.1007/s00592-018-1119-x

**Published:** 2018-02-17

**Authors:** Mikaela B. von Bonsdorff, Monika E. von Bonsdorff, Maija Haanpää, Minna Salonen, Tuija M. Mikkola, Hannu Kautiainen, Johan G. Eriksson

**Affiliations:** 1 Gerontology Research Center, Faculty of Sport and Health Sciences, University of Jyväskylä, PO Box 35, 40014 Jyväskylä, Finland; 2 Folkhälsan Research Center, Helsinki, Finland; 3 Helsinki University Central Hospital, Helsinki, Finland; 4 Etera Mutual Pension Insurance Company, Helsinki, Finland; 5 Department of Chronic Disease Prevention, National Institute for Health and Welfare, Helsinki, Finland; 6 Department of General Practice and Primary Health Care, University of Helsinki and Helsinki University Hospital, Helsinki, Finland

**Keywords:** Diabetes mellitus, Diabetes medication, Early exit from workforce, Retirement, Disability pension, Life course, Epidemiology, Aging

## Abstract

**Aims:**

Early exit from the workforce has been proposed to be one of the unfavorable consequences of diabetes. We examined whether early exit from the workforce differed between persons who were and were not diagnosed with diabetes during their work career.

**Methods:**

The cohort included 12,726 individuals of the Helsinki Birth Cohort Study, born between 1934 and 1944. Using data from nationwide registers, the cohort was followed up from early adulthood until they transitioned into retirement or died. Work-loss years were estimated using the restricted mean work years method.

**Results:**

During a follow-up of 382,328 person-years for men and 349 894 for women, 36.8% transitioned into old age pension and 63.2% exited workforce early. Among men, 40.5% of those with and 32.8% of those without diabetes transitioned into old age pension (p=0.003). The corresponding numbers for women were 48.6% and 40.4% (*p* = 0.013), respectively. Mean age at exit from the workforce was 60.1 (95% confidence interval [CI], 59.6 to 60.7) years among men with diabetes and 57.6 (95% CI, 57.2 to 58.0) years among men without diabetes (*p* = 0.016). Among women, corresponding ages were 61.4 (95% CI, 60.8 to 61.9) years for those with diabetes and 59.5 (95% CI, 59.3 to 59.7) years for those without diabetes (*p* < 0.001). The difference in mean restricted work-loss years according to diabetes was 2.5 (95% CI 0.5 to 4.6) for men and 1.9 (95% CI 1.0 to 2.8) for women.

**Conclusion:**

Among individuals followed up throughout their work career, those with a diabetes diagnosis exited the workforce approximately two years later compared to those without diabetes.

## Introduction

Governments’ long-term economic projections for public expenditure on pensions are strongly reliant on effective retirement ages and the assumption that people will exit the workforce at an older age in the future [1]. The employment rates of people aged 55–64 years have been rising during recent decades in the USA [2] and in the OECD countries, in the latter ones from 52 to 57% on average [3]. This is partly due to pension reforms in several countries. In 2014, the average pension age in OECD countries was 64.6 years for men and 63.1 years for women [3]. However, for example in Finland, in the year 2011 almost one-third of the working aged people had transitioned into disability pension already at an average age of 52 years [4]. Early exit from the labor market is a challenge for the societies and has severe economic implications.

Diabetes is globally one of the main non-communicable diseases with a prevalence of 422 million cases totaling 8.5% of the adult population in 2014 [5]. As diabetes places a notable burden on the society [6], it can be viewed as a public health disease and an indicator of the general cardio-metabolic health status of the population. Negative impacts of diabetes on direct costs such as healthcare services and indirect costs such as loss in productivity in, e.g., early exit from the workforce have been reported [7]. However, some inconsistent findings on the association between diabetes and premature retirement have been reported. In a 2-year follow-up in the US Health and Retirement Study, diabetes was found to increase the risk of work cessation compared to those with no diabetes among middle-aged men but not among women [8]. During an 18-year follow-up in the French GAZEL cohort, people with diabetes showed a trend toward an increased probability of transitioning from employment to work disability including disability pension compared to non-diabetics [9].

The association between diabetes and early retirement has so far not been studied from a life course perspective covering the entire work career. Using data from the Helsinki Birth Cohort Study, we studied whether early exit from the workforce differed between persons who were and were not diagnosed with diabetes during their work career.

## Materials and methods

### Study population

The Helsinki Birth Cohort Study comprises 13,345 individuals born in Helsinki, Finland, at Helsinki University Central Hospital or Helsinki City Maternity Hospital between 1934 and 1944 [10, 11]. The unique personal identity number assigned to all Finnish residents was used to link data from several national registers. The retirement decisions and records of diabetes date back to 1964 (cohort members were 20–30 years old), and they were followed up until the end of year 2013 (cohort members were 69–79 years old). Of the original cohort, we excluded those who had missing data on retirement (*n* = 112) and those who had migrated before retirement (*n* = 507). Thus, our study cohort included 12,726 individuals who were followed up from early adulthood when they were available for work until they exited the workforce or died. The study was approved by the Ethics Committee of Epidemiology and Public Health of the Hospital District of Helsinki and Uusimaa and that of the National Public Health Institute, Helsinki.

### Early exit from the workforce

Information on date and type of pension was provided by the Finnish Centre for Pensions and the Finnish Social Insurance Institution and date of death by the Finnish Population Register Centre and was available for all cohort members. All Finnish residents who have lived in Finland for at least 3 years after they turned 16 years are covered by the pension system and are thus included in the pension registers. In Finland until 2005, the statutory retirement age was 63–65 years and from 2005 to 2016 63–68 years with the exception of certain professions that had younger retirement ages such as firemen and pilots. In the present study, the cohort members either transitioned into old age pension at the respective statutory retirement age or retired earlier and transitioned into disability, unemployment or part-time pension or died before retiring. Around 5% of the cohort carried on working past the age of 65 years (e.g., entrepreneurs working in the private sector). For these cohort members, there are no retirement data available in the pension registers during the follow-up. Among those who transitioned into *old age pension*, 90% retired at age 58–65 years. They did not have any work-loss years and were considered to have completed a normal work career. *Early exit from the workforce* in this study meant that the person transitioned into retirement before statutory pension age due to disability, unemployment or part-time pension or died before retiring. Disability pension is granted due to a medically confirmed illness when a person is unable to continue working even after periods of rehabilitation, re-education or assistance. For qualifying for unemployment pension, one needs to be unemployed, at least aged 60 years, had received unemployment benefits for at least 500 days and had accumulated employment pension for at least 5 years during the past 15 years. Part-time pension meant that the person was partly working and partly retired. We calculated the work-loss years for each cohort member who retired early (due to disability, unemployment or part-time pension or death before any retirement) by deducting the date of retirement from the date when the cohort member turned 65 years.

### Ascertainment of diabetes

Identification of diabetes cases was based on a record from any of the three sources: (1) record of any kind of diabetes medication purchase from the nationwide prescription register of the Social Insurance Institution (Anatomical Therapeutic Chemical [ATC] codes A10A, A10B and A10X) available from year 1995 onwards; (2) a record of entitlement to reimbursement of medicine costs for diabetes from the Social Insurance Institution (code 103) available from year 1964 onwards and (3) in- or outpatient care record with ICD-9 code 250 or ICD-10 code E10, E11, E12, E13 or E14 from the national Care Register for Health Care available from year 1969 onwards. Each person was followed up for a record of diabetes until they transitioned into retirement or died.

### Socioeconomic status

Socioeconomic status in adulthood was ascertained using register data from Statistics Finland at 5-year intervals between the years 1970 and 2000. Highest occupational status was coded as upper middle class, lower middle class, self-employed and manual workers [12]. Highest educational attainment was ascertained using register data from Statistics Finland and coded as basic/primary or less, upper secondary, lower tertiary and upper tertiary.

### Statistical analyses

Characteristics of the cohort members were compared according to diabetes status using *t* test for continuous variables and Chi-square test for discrete variables. The cumulative percentage and differences in estimates of work-loss years across the work career according to diabetes status were estimated for men and women and illustrated by the Kaplan–Meier method. Differences between cumulative incidence curves were estimated using Cox proportional hazards regression analyses with 5-knot restricted cubic splines adjusted for fixed covariate levels (birth year and socioeconomic status in adulthood). Average age of exit from the workforce and the difference according to diabetes status was estimated using the restricted mean “survival” (work years) time (RMST) method [13]. All tests were performed two-tailed, the level of significance was set at *p* < 0.05 and analyses were carried out with STATA 14.1 (StataCorp LP, College Station, TX).

## Results

The men in the cohort were followed up for 382,328 and the women for 349,894 person-years from early adulthood until they retired or died. Of the cohort members, 52.9% were men and the social class distribution of the cohort members was laborer for 40.4%, self-employed for 9.9% and low or high official for 49.6%. Of the cohort members, 36.8% had transitioned into old age pension and had no work-loss years. The rest (63.2%) retired early or died prematurely—28.8% transitioned into disability pension, 14.0% into unemployment pension, 12.8% into part-time pension and 7.6% had died before transitioning into any form of retirement. Of the 12,726 cohort members, 716 (469/6259, 7.5% men and 247/5751, 4.3% women) had a record of diabetes before they retired or died without first transitioning into retirement. The mean age at first record of diabetes was 54.5 (SD 6.5) years for men and 54.7 (SD 7.0) years for women. Among women, childhood socioeconomic status was lower for those who had a record of diabetes (*p* = 0.02) and among men a similar trend was observed for socioeconomic status in adulthood (*p* = 0.06). There were no differences in educational attainment according to diabetes status.

Table 1 presents retirement events and mean ages according to diabetes status. Among men, 40.5% of those with and 32.8% of those without diabetes transitioned into old age pension (*p* = 0.003). Among women, 48.6% of those with and 40.4% of those without diabetes transitioned into old age pension (*p* = 0.013). Men and women with diabetes who transitioned into disability pension or died before retirement were older than those without diabetes (*p* < 0.001).

**Table 1:** Retirement events/deaths before retirement (number, proportion) and age in years (mean, SD) according to diabetes status

	Retirement events			Age at retirement		
	Diabetes	No diabetes	*p*	Diabetes	No diabetes	*p*
Men						
Old age pension	190 (40.5)	2050 (32.8)	0.003	63.1 (1.9)	62.2 (3.4)	< 0.001
Premature pension						
Disability	128 (27.3)	1861 (29.7)		56.0 (5.5)	51.6 (8.5)	
Unemployment	54 (11.5)	892 (14.3)		60.2 (0.8)	60.1 (0.9)	
Part-time	61 (13.0)	769 (12.2)		58.4 (2.2)	58.3 (2.1)	
Death before retirement	36 (7.7)	687 (11.0)		51.3 (8.3)	47.4 (8.9)	
Women						
Old age pension	120 (48.6)	2326 (40.4)	0.013	62.7 (2.1)	62.3 (2.4)	< 0.001
Premature pension						
Disability	49 (19.8)	1626 (28.3)		56.0 (8.1)	52.8 (7.6)	
Unemployment	41 (16.6)	796 (13.8)		60.4 (1.3)	60.1 (0.95)	
Part-time	26 (10.5)	769 (13.4)		58.4 (1.9)	58.2 (1.9)	
Death before retirement	11 (4.5)	234 (4.1)		51.5 (10.1)	49.9 (8.6)	

**Fig. 1:**
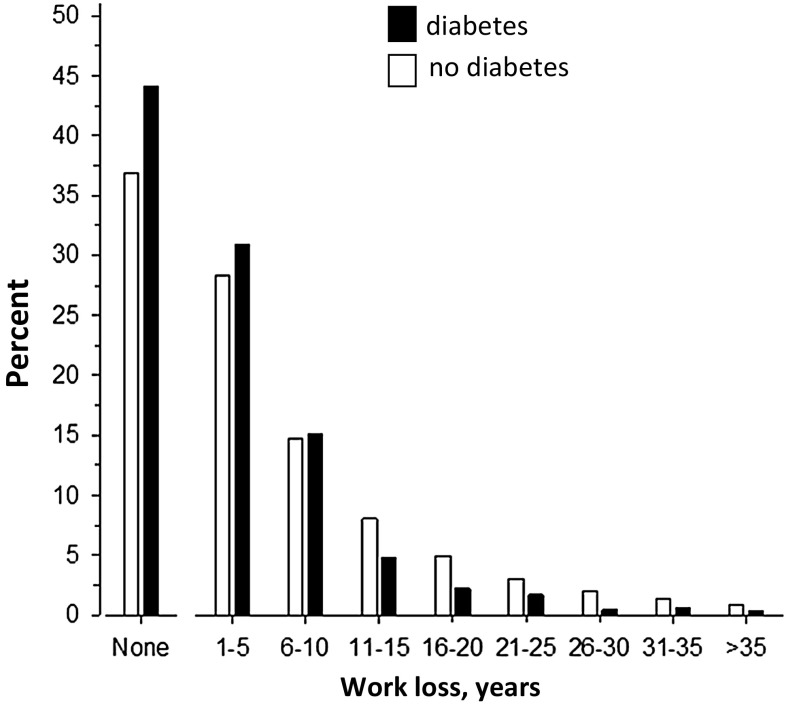
Distribution of work-loss years during the work career for all cohort members according to diabetes status

Figure 1 shows the distribution of work-loss years across the work career for all cohort members with and without diabetes. Of the subjects with diabetes 44.1% and of those without diabetes, 36.9% had no work-loss years, i.e., they transitioned into old age pension. The number of work-loss years varied between 1 and 44 years in the whole cohort. There were more persons with than without diabetes who had at most five work-loss years 30.9 versus 28.3%.

**Fig. 2:**
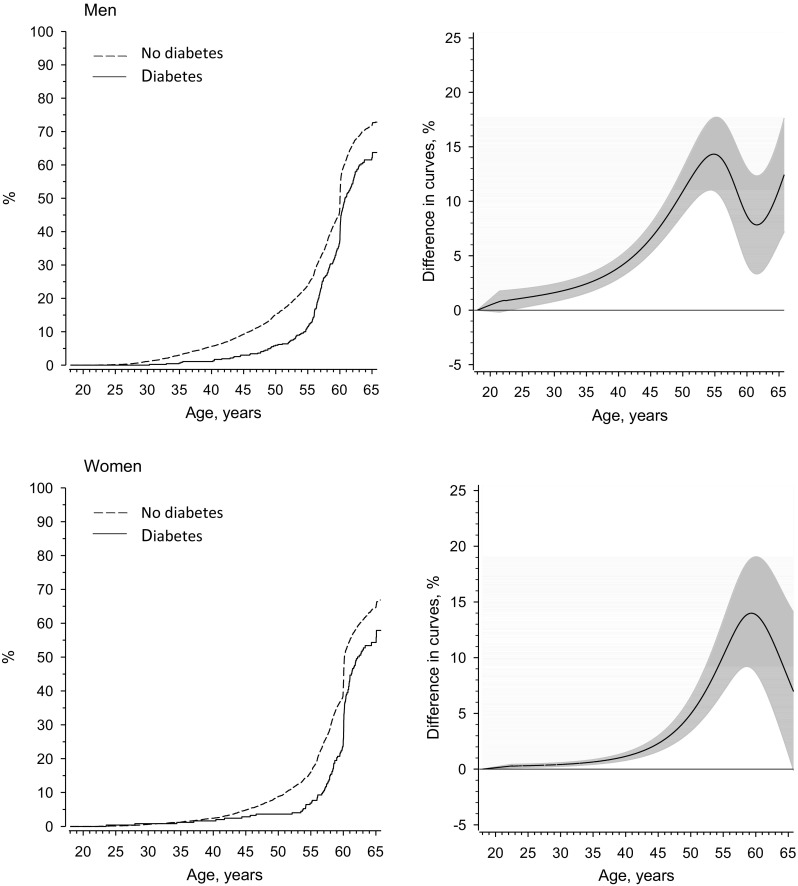
Left panel: the risk of work-loss increasing age according to diabetes status for men and women who existed workforce early. Right panel: The difference in the curves were derived from 5-knot (age centiles: 17, 33, 50, 67 and 83) restricted cubic spline regression models. The models were adjusted for birth year and adult socioeconomic status. Gray area represents 95% confidence intervals

Figure 2 shows the risk of work-loss years with increasing age according to diabetes status for men and women who exited the workforce early and the differences in proportions of work-loss. For men the lines start to diverge already at age 30 years according to diabetes status. A similar trend was observed among women, i.e., the lines started to diverge after age 40 years. The difference in work-loss years according to diabetes status was biggest at age 55 years for men and 58 years for women. Among men, the mean retirement age was 60.1 (95% CI 59.6–60.7) years for those with and 57.6 (95% CI 57.2–58.0) years for those without diabetes (*p* = 0.016, adjusted for birth year and adult socioeconomic status). Among women, the mean retirement age was 61.4 (95% CI 60.8–61.9) years for those with and 59.5 (95% CI 59.3–59.7) years for those without diabetes (*p* < 0.001 adjusted for birth year and adult socioeconomic status). The difference in the mean restricted work years according to diabetes status was 2.5 (95% CI 0.5–4.6) for men and 1.9 (95% CI 1.0–2.8) for women in favor of those who had a record of diabetes during their work career.

## Discussion

In this birth cohort followed up from early adulthood until retirement or death, we found that the men and women who had a diabetes diagnosis exited the workforce approximately 2 years later compared to those without a record of diabetes. The difference in work-loss years between those with and without diabetes started to diverge already at age 30 years for men and at age 40 years for women and was higher for those with no record of diabetes. We investigated the main types of workforce exit including transitioning into old age, disability, unemployment and part-time pension as well as premature death.

In general, diabetes has been found to be related to loss in productivity in, e.g., early exit from the labor market [7]. However, studies have so far mostly reported cross-sectional findings or have had relatively short follow-up times. Ervasti et al. [14] found in a Swedish register-based study that those who were diagnosed with diabetes had a higher number of annual work disability days around the time of diabetes diagnosis during the 7-year study period. The study outcome included both sickness absence and disability pension days, and it is not evident what the proportion of disability pension days was for those with incident diabetes during the 7-year follow-up. The study by Herquelot et al. [9] using data from the GAZEL cohort is one of the few studies which has a longer follow-up time. In the 18-year follow-up, the hazard of transitioning from employment to retirement or death for those with diabetes compared to those without diabetes was higher. However, in the study by Herquelot et al. [9] the hazard for transitioning into disability pension or being on sick leave for at least 1 year for those with diabetes compared to those without diabetes attenuated after adjustment for body mass index and was not statistically significant any longer. These findings together with our present findings provide some evidence that the association between diabetes and early workforce exit may not be as persistent as previously reported in studies based on shorter surveillance time.

There are several potential factors that might explain our finding that those with diabetes had less work-loss years than those without diabetes. Although it has been shown that individuals who had a record of diabetes were more likely to be in worse health than those who did not have a record of diabetes, they might have received effective medical care for the disease and related conditions [5, 15], thus potentially contributing to better overall health status and work ability [16]. Diabetes research has a long tradition in Finland. For example, the study findings of Yki-Järvinen et al. [17] in early 1990s on insulin therapy in type 2 diabetes has received a lot of international interest and the findings largely influenced the Finnish treatment strategies. Recent guidelines on diabetes care recommend simultaneous treatment for other closely related conditions such as dyslipidemia [18]. As part of standard treatment, diabetics receive lifestyle counseling which is likely to increase their awareness of the risks related to unhealthy living habits such as smoking which have been shown to be related to earlier exit from working life [19]. There is a growing body of evidence showing that interventions to prevent and control diabetes are successful and that they are cost-effective [20]. For example, a recent randomized trial with an 8-year intensified multifactorial intervention including behavioral and pharmacological approaches versus standard treatment among diabetics increased the median survival time and time free of cardiovascular disease by an average of 8 years [21]. Such findings, along with other recent positive results from trials on new diabetes drugs [22], indicate that the impact of diabetes on work career performance might be effectively mitigated through multifactorial targeted interventions.

The prevalence of diabetes was 5.6% in our cohort. This is in line with the 4.5% of Finnish residents aged 15–65 years who had a record of diabetes medication (ATC code A10) purchase in the prescription register of the Social Insurance Institution in the year 2016. However, under-diagnosis of diabetes is common and estimated to be around 20–50% [23]. It is likely that there are a number of individuals in the workforce suffering from undiagnosed and untreated diabetes who perform worse than their healthy peers.

The strengths of our study include the long follow-up time covering the work career from early adulthood to retirement (or premature death). We were able to follow up the cohort until 95% of the cohort transitioned into retirement or died prematurely. Earlier evidence on the relationship between diabetes and retirement comes mainly from cross-sectional studies which might be biased in terms of representativeness of the study population [24]. Our data originate from the database of the nationwide social security system which has data on income security and social healthcare services, and include objective data and have been validated and found to be reliable [25]. Register data on pensions were available for all original cohort members (except for those who died or migrated before retirement), thus minimizing selection bias related to loss to follow-up. We had information on adult socioeconomic status from the national register for the entire cohort. The purpose of this paper was to investigate those with a diabetes diagnosis as an entity including other related comorbidities, which are known to be related to diabetes [14]; thus, we did not adjust for other chronic illnesses.

Some limitations of the study should be recognized. First, we focused on work-loss at the end of the work career. Diabetes may have resulted in sickness absence days during the work career, but we did not have these data available and cannot estimate the impact of diabetes on absence from work [26]. Second, specific information on type 1 and type 2 diabetes was not available. Based on detailed assessments of the clinical sample of the HBCS data, about 5% of diabetes cases were type 1 and the rest type 2 diabetes. We do not have information on the exact date of diabetes diagnosis, but have used the first record of a diabetes medication reimbursement decision and/or purchase of medication and/or hospital care due to diabetes as the onset day of diabetes. Furthermore, we did not have data available on, e.g., work strain, work ability or lifestyle factors that might have explained some of the association analyzed here [27]. This is often the case in studies, so residual confounding might occur.

In conclusion, we found that persons who had a record of diabetes exited the workforce approximately two years later compared to those who did not have a record of diabetes. This study is to our knowledge the first that includes data across the entire work career for men and women. We also included premature death into our workforce exit events. Our finding is of major importance as the older population is encouraged to complete longer work careers and supports the importance of optimal medical and particularly diabetes treatment.
